# “They need more programs for the kids:” Low-income mothers’ views of government amidst economic precarity and burdensome programs

**DOI:** 10.1111/ijsw.12641

**Published:** 2024-01-21

**Authors:** Carolyn Y. Barnes, Sarah Halpern-Meekin, Jill Hoiting

**Affiliations:** 1 Crown Family School of Social Work, Policy, and Practice, University of Chicago, Chicago, Illinois, USA; 2 School of Human Ecology and La Follette School of Public Affairs, University of Wisconsin, Madison, Wisconsin, USA; 3 Sandra Rosenbaum School of Social Work, University of Wisconsin, Madison, Wisconsin, USA

**Keywords:** administrative burden, collective motherhood, government responsiveness, policy feedback, qualitative research

## Abstract

Policy feedback scholars argue that experiences with government shape political participation. Administrative burden scholars posit that burdensome bureaucratic encounters deter political participation. Related quantitative studies take a top-down, deductive approach and test effects of single policies, yet people engage multiple programs, and all policies may not be equally salient in how they view the state. Using qualitative interviews, our inductive, “bottom-up” approach examines the most prominent policy domains in views of government shared across mothers with low incomes in the United States (*n* = 80). Mothers experienced a wide range of policies, and they detailed related administrative burdens, but this was not the focus in most of their views of the government. Many raised issue areas that hit close to home, such as affordable child care or children’s recreation programs. They often drew on a sense of collective motherhood in their view of government, including how it could facilitate efforts to raise children.

## INTRODUCTION

In recent years, scholars have increasingly turned their attention to the ways that “policy administration shapes politics” (Moynihan & Soss, 2014, p. 1). There is growing recognition that bureaucracies “can reorganize power relations in a society, redefine terms of political conflict, mobilize or pacify constituencies, and convey cues about group deservingness” (Moynihan & Soss, 2014, p. 3). In doing so, these bureaucratic encounters, or administrative burdens, can shape people’s views of, demands on, and engagement with the government.

This connection between policy and politics is not new. Scholars have long argued that “new policy creates new politics” (Schattschneider, 1935, p. 288) and shown how policy creates feedback effects, informing the development of interest groups and the behavior of political elites and mass publics (Pierson, 1993; Skocpol, 1992). Other studies probe how policies shape everyday citizens’ willingness and capacity to participate politically (Campbell, 2003; Goss et al., 2019; Lerman & Weaver, 2014; Mettler, 2005; Michener, 2018; Rhodes, 2015; Rose, 2018; Soss, 2000). Public policies can incentivize political behavior and build capacity for civic and political engagement or act as sources of information and meaning for citizens (Campbell, 2012). Scholars of administrative burden—onerous experiences with policy implementation—have called for research on how costly bureaucratic encounters shape dimensions of democratic citizenship. For example, Baekgaard and Tankink (2022) call for research that examines the extent to which “experiences of administrative burden mediate the impact of state actions on citizen outcomes, policy take-up, civic participation, and political participation” (p. 19).

Yet, with a few exceptions, both the policy feedback and administrative burden perspectives take an “elite centered” rather than a “bottom up” and “citizen-centered” view of how individuals experience policy, meaning they focus on those policies and features that those in positions of power select. This includes key assumptions the feedback perspective makes about how citizens interpret policies. In the past, policy feedback studies have left little room to examine how individuals construct their own political identities independent of the social constructions imposed by public policy (Schneider & Ingram, 1993). However, newer research suggests that individuals bring their own social identities to how they interpret their experiences with government, support public policies, and engage in political participation (Barnes, 2020; Mettler, 2018; Nuamah, 2022).

How individuals understand public policy might reflect a “group-based perspective” rooted in their social identities (Walsh, 2012) and constructed from the “bottom-up” by people themselves rather than from social constructions imposed by policies. While convincing evidence shows how policies can create political constituencies (Mettler & Soss, 2004), program beneficiaries may construct their political identities beyond the narrow boundaries of a Medicaid or TANF recipient, for example. Further, political identities are more complicated than the independent effects of each of a person’s background characteristics. Rather, how clients interpret policy may result from nuanced constructed identities that serve as the lens through which clients make sense of their political world (Soss, 2005). Michener et al. (2022) also raise this point and call for “bottom-up” research that shifts what researchers attend to when examining the welfare state, putting individuals’ perspectives front and center in the analysis. They call for research that diverges from “research determined measures” and hypotheses to include inductive qualitative research that seeks to understand policy through the eyes of those who experience it (Michener et al., 2022, p. 165).

Regarding administrative burden research, scholarship similarly imposes “elite-produced” views of how people experience policies and how these experiences may matter for political participation (Michener et al., 2022, p. 164). Much like policy feedback studies, administrative burden research focuses on a single policy first, then asks policy targets to describe their experiences with this policy (Barnes, 2021; Barnes & Henly, 2018; Bell et al., 2023; Heinrich, 2016; Herd et al., 2013; Nisar, 2018). For example, Barnes and Henly (2018) probe the bureaucratic encounters parents have in the Child Care Subsidy program; Bell et al. (2022) examine burdensome experiences in a means-tested tuition-free college program; Masood and Nisar (2021) demonstrate how doctors apply for maternity leave; and Baekgaard and colleagues examine experiences with the Danish Social Benefit program (Baekgaard & Madsen, 2023; Madsen et al., 2023).

This approach runs counter to how people actually experience policies; as Campbell (2012) notes, “individuals live in a world of multiple jurisdictions and are affected by multiple policies at once” (p. 347). Marginalized families likely engage with multiple means-tested programs from a range of public and private providers (Allard, 2009). In addition, public policies intersect with people’s everyday lives beyond direct experience with programs. Recipients of means-tested programs live in communities, have families, and are employed—aspects of everyday life that intertwine with policy. Thus, feedback effects “depend ultimately on how public policies fit into the lives of individuals and social groups” (Mettler & Soss, 2004, p. 64).

With some exceptions, most administrative burden research is deductive. Recent reviews of the administrative burden research suggest that the field’s “understanding of people’s experiences is still very much based on the deductive categorization of experiences” (Halling & Bækgaard, 2023, p. 31). As a consequence, researchers seldom ask individuals to describe how the “state” fits into their lives, how they make claims on the state, and how they construct their own views of government. Consequently, the existing lines of research on administrative burden and policy feedback often do not give room for how individuals may experience multiple programs at once or have distinct views on whether and how burdensome experiences matter for how they think and act politically.

Using qualitative interviews with 80 mothers with low incomes and young children, we build on the intersection of the policy feedback and administrative burden frameworks to examine which policy domains are most salient to them. We ask, what dimensions of public policy inform mothers’ views of government responsiveness? How do mothers draw on their experiences with public assistance programs as they express their views of government? How do they construct political identities when making sense of government responsiveness, otherwise known as external political efficacy (Niemi et al., 1991)?

The contributions of this study are three-fold. First, we meet the call for “citizen-centered,” “in-depth work” that treats individuals as active agents in feedback processes and uncovers “the subjective perceptions and knowledge of individuals” instrumental to feedback processes (Campbell, 2012, p. 347). We demonstrate mothers’ interpretive processes—how they consider policy when discussing views of government, which aspects of policy convey responsive government in their eyes, and how they develop and use a collective identity to interpret policy outcomes.

This analysis pushes both the administrative burden and policy feedback research literatures to carefully consider the lived experiences of “policy targets” and how policy intersects with their day-to-day lives to inform their political identities and views of the state. We find that, despite sharing narratives describing administrative burdens with a range of public assistance programs, mothers did not commonly refer to onerous bureaucratic encounters when asked to describe their views of government. Instead, mothers emphasized the need for more generous child-targeted programs, access to recreational activities, and quality schools when assessing government responsiveness and articulating their claims on government. These demands extended beyond their bureaucratic encounters with government programs. Further, these perspectives demonstrate how intersecting child and family policies cultivate a collective political identity around motherhood.

### Policy feedback

Policy feedback scholars posit that the design of public policies can shape mass political behavior by equipping individuals with the resources to participate (resource effects) or teaching lessons about the government and political standing that shape interests in political participation (interpretive effects) (Pierson, 1993). With regard to resource effects, policies can increase access to resources like time, money, civic skills, and opportunities for recruitment into political participation. For example, Mettler (2005) demonstrates how the GI Bill boosted civic engagement among veterans from low to moderate socioeconomic backgrounds by expanding access to higher education. The education benefits of the GI bill provided financial resources, inculcated civic skills, and created social networks that could facilitate recruitment into civic and political engagement. Similarly, Rose (2018) shows how legislation (e.g., National Defense Education Act of 1958, Higher Education Act of 1965, and Title IX of the 1972 Education Amendment) increased access to higher education for women, boosting political engagement. Other research shows how policies create constituencies by increasing their material stake in politics. Campbell’s (2003) work show-s how Social Security politically mobilized senior citizens by elevating their material stake in the policy’s survival.

Along with providing resources for political participation, policies can convey important lessons about the government and political standing—interpretive effects (Pierson, 1993). Citizens learn these messages broadly through the media and mass communication or through direct experience with policy designs (Schneider & Ingram, 1993; Soss, 1999a). Policy designs are imbued with social constructions—cultural characterizations and images associated with policy targets—that convey the policy targets’ normative valence and political influence (Schneider & Ingram, 1993; Schneider & Sidney, 2009). Citizens may consider how policies affect the general public, relying on media and mass communication to forge attitudes about policies and government (Jacobs & Mettler, 2020; Jacobs et al., 2022). As citizens engage policies and experience policy implementation, they can internalize these social constructions and learn their political status in relation to the government and other groups. These messages, in turn, influence how targets think and act politically. For example, scholars attribute low levels of political engagement among clients of public assistance programs to their negative experiences with means-tested programs (Soss, 2000; Verba et al., 1995). Programs such as Aid to Families and Dependent Children (AFDC) or Temporary Assistance to Needy Families (TANF) frame targets as welfare dependents or deviants that should be punished or controlled (Michener, 2019; Schneider & Sidney, 2009; Soss, 1999a). The intrusive, stigma-laden intake processes teach recipients that the government is an autonomous directive force that is unresponsive to their interests (Bruch et al., 2010; Schneider & Ingram, 1993; Soss, 1999b). In the same way, contact with the authoritarian carceral state similarly depresses political engagement (Garcia-Rios et al., 2021; Lerman & Weaver, 2014).

With some important exceptions (Barnes, 2020; Michener, 2018; Nuamah, 2022; Soss, 2000, 2005), most policy feedback studies are quantitative and measure the effects of program participation on political behavior (Bruch et al., 2010; Campbell, 2003; Mettler, 2011). As a result, policy feedback research is limited in the insights it can offer into the interpretive process that leads disadvantaged citizens to their sense of external political efficacy and broader views of government.

### Administrative burden and policy feedback

Administrative burden refers to individuals’ onerous experiences with policy implementation (Burden et al., 2012). Drawing from interdisciplinary studies of social policy, Moynihan et al. (2015) identify three kinds of costs individuals incur during policy implementation: the cost of learning about programs and how to apply (learning costs); the cost of abiding by program rules (compliance costs); and the stress and stigma of applying for and using benefits (psychological costs).

Qualitative studies have demonstrated how individuals experience these costs. Earlier work shows the stigmatizing nature of intake processes for means-tested programs (Hays, 2004; Soss, 1999a; Watkins-Hayes, 2011), and more recent work reveals how claimants experience psychological and compliance costs of programs (Barnes & Henly, 2018); Barnes (2020) illustrates a subset of learning costs—redemption costs or learning how to use benefits. Even so, administrative burden research is limited in showing how costly experiences with the state shape views of government. Studies that do take up this question have not yet examined how policy targets or beneficiaries learn political lessons from burdensome experiences with the state. To be sure, administrative burden researchers defer to earlier policy feedback research as evidence of the political consequences of costly experiences with the state (Moynihan, 2022). Further, recent reviews of the literature push scholars to consider questions about whether “burdensome encounters with the state have further detrimental effects on citizens’ lives and democratic behaviors” and “which kinds of state actions have the most detrimental consequences for people’s lives and democratic participation” (Halling & Bækgaard, 2023, p. 31).

Administrative burden studies that do draw attention to forms of policy feedback have focused on how awareness of burdens in targeted programs shapes support for these programs among the public and elected officials. For example, Keiser and Miller (2020) find that information about the administrative burden of TANF in eligibility boosts citizens’ approval of this program. Other research demonstrates burden tolerance—the willingness of policymakers to allow or impose administrative burdens (Baekgaard et al., 2021; Halling et al., 2023).

Scholars cast burdens as deliberate ideological and partisan tools to obstruct program participation (Herd et al., 2013; Moynihan et al., 2016). Moynihan et al. (2016) find that states with unified Democratic party control have less burdensome Medicaid programs—shorter application times and fewer document requirements. In contrast, conservative politicians are more tolerant of administrative burdens but are prone to imposing fewer burdens on policy targets that are viewed as deserving (Baekgaard et al., 2021). Taken together, these findings help reveal views of burdens among elites and the general public but offer fewer in-depth insights into how policy targets construct broader views of government and the extent to which burdensome experiences with the state shape those views.

## METHODS

We use data gathered over multiple waves of semistructured interviews in the Baby’s First Years: Mothers’ Voices (BFY: MV) study, which is a qualitative companion to the larger randomized controlled trial, Baby’s First Years (BFY). This RCT examines the impact of unconditional cash transfers provided to mothers with limited incomes on their child’s development. While in the hospital after giving birth, 1000 mothers were recruited across four cities: New Orleans, New York City, Omaha, and the Twin Cities. These metropolitan areas were selected because of their varying policy contexts and demographic compositions. Upon agreeing to participate in the BFY study, mothers were randomly assigned to receive a monthly cash gift of either $333 or $20 via debit card for the first 76 months of their child’s life (for details of the RCT study design, see Gennetian et al., 2023 and Noble et al., 2021). All mothers were 18 or older, had incomes below the federal poverty line, and had babies who did not need care in the neonatal intensive care unit.

The 80 mothers in BFY: MV were randomly selected from two of the four BFY sites: New Orleans and the Twin Cities, with the sample stratified to match proportions of BFY mothers in each site, equally include mothers receiving the large and small cash gifts, and ensure representation of first-time mothers. Most of the mothers in BFY: MV are women of color. Sixty-six percent as Black, 9% as Hispanic, 4% as Asian, 1% as American Indian, 10% as white, and 10% identify with multiple races or another race. At the time of the first wave of interviews, mothers’ ages ranged from 19 to 42, with a median age of 27. At this time, BFY: MV mothers had between one and six children, with a median of two; 29% were first-time mothers. Forty percent of mothers lived with a romantic partner and 31% lived with a family member of an older generation (Table 1).

In this study, we draw on data from four waves of BFY: MV interviews and an interim wave of interviews with mothers in New Orleans conducted to capture experiences during the COVID-19 pandemic. We have interviewed 98% of mothers at least twice across the four waves.^1^ We first interviewed mothers when their children were between 10 and 21 months old (13 months old, on average). Subsequent waves of interviews occurred, on average, about every 10 months, except for the pandemic-specific interviews which were staggered around the second wave of interviews, during the first year of the pandemic. While our first wave of interviews began in-person, we pivoted to phone interviews in response to the COVID-19 pandemic which continued for subsequent waves. The interviews covered a broad range of topics about mothers’ lives and experiences in managing parenthood, work, and finances. Over the course of the interviews, 65% of mothers were formally employed at some point. Across interview waves, we asked mothers about their sources of income and experiences with income support programs. Mothers were using or had previously used a variety of programs including SNAP (at least 88% of mothers), WIC (at least 94% of mothers), housing assistance (e.g., Section 8, public housing; at least 54% of mothers), early care and education assistance (e.g., child care subsidies, Head Start; at least 33%), and TANF (at least 33% of mothers).^2^ In addition, many mothers had present or past experiences with Medicaid, and other mothers had experiences with programs such as unemployment insurance and those through the Social Security Administration (e.g., supplemental security income, survivor benefits). We also asked about their experiences of and perspectives on motherhood, including both the best and most stressful parts. Particularly pertinent to the present study, in the third wave of interviews, we explicitly asked mothers, “What could the government do to help families with young children like yours?” All interviews were recorded and transcribed.

We take both deductive and inductive approaches in our analysis. Using a pre-determined set of descriptive codes for each wave of interviews, we tabulated whether mothers had previous or current experience with various income support programs. We also counted how many mothers shared at least one story of administrative burdens she had experienced. We asked generally about experiences with public benefit programs at each wave; then, during the fourth wave, we also asked mothers about application processes and any benefit delays experienced. Such daily hassles or difficult program interactions are one way administrative burdens manifest on the ground for mothers. Though we did not specifically ask about administrative burdens until this later interview, most mothers had described experiencing burdens in earlier interviews, whether those were compliance, learning, or psychological in nature. Using *Dedoose 9.0*, we applied a set of deductive codes to transcripts, covering numerous topics which evolved over time as new waves of interviews encompassed new subject matter, examples of codes include Income Sources, Income Support Program Experiences, Stressful Parts of Motherhood, Best Parts of Motherhood, and Views of Government. We followed a coding consistency protocol to ensure that coders conceptualized and applied codes similarly. At regular intervals, two coders independently coded a transcript and then compared and reconciled their coding in cooperation with a third member of the research team. Drawing on excerpts from interviews, we then analyzed trends and identified themes that emerged from the ways in which mothers discussed their experiences with and views of government. We use pseudonyms for all mothers to protect their privacy.

## RESULTS

### External efficacy

Mothers reported distinct views of external efficacy, or government responsiveness that reflected areas and interventions where they saw government responses as desirable and possible. Mothers emphasized additional government action and provision within the confines of existing systems. For example, they asked for more help with child care rather than the creation of paid parental leave or for more housing assistance rather than unconditional cash transfers, like BFY, that mothers could use as they desired.

Mothers expressed expectations for government responsiveness through greater resources (described by 28 mothers), child activities (11 mothers), and better schools (8 mothers). Notably, only six mothers named eased administrative burdens in targeted programs as a way the government could better respond to families. Only one or two mothers discussed other areas for government responsiveness.^3^

A small number of mothers did not expect more from the government because (1) they felt that what they were receiving was adequate, (2) it was not a question they felt they knew how to answer, or (3) they did not trust the government to do anything for them. As an example of the first, Star, a Black mother in the Twin Cities, said:

I don’t know. I think they’re doing a pretty decent job. I can’t really complain because I’m working now, everything is okay for me, but someone else’s situation might be harder because maybe they don’t have child care or anything like that. But for me...from my personal experience, I think they’re doing okay.

As an example of the second, Fatima, a North African mother in the Twin Cities, said, “I don’t know. I don’t have any idea about that. I didn’t like, I never looked through that. I don’t have no idea.” And as an example of the third, Bianca, a Black mother in New Orleans, said, “I can’t really say. I don’t know. I’m not really, like, into the government because I don’t think it’s for everybody. … They lie a lot so I really wouldn’t have an answer.” Most mothers neither expressed Star’s satisfaction nor Fatima’s lack of opinion. Importantly, most mothers did not reject the premise of the question. Unlike Bianca, they conveyed distinct external political efficacy beliefs and views on social inclusion when asked to describe what the government could do for them.

Mothers most often described categories of government responsiveness focused on greater resources to support core responsibilities of parenthood and child-related needs and activities. The far most common was a desire for more resources of various kinds. These reflect a desire for government assistance in the areas core to parenthood—a request for recognition of the work and daily realities of parenthood through the provision of support for these activities.

Krista, a white mother in New Orleans, explained that being able to find affordable child care would mean she could work during the day, rather than needing to work at night, when someone else was available to care for her children:

I kind of wish that they wouldn’t be so, like, strict on, like, child care assistance, because child care is expensive. And I really think that that’s something like they should offer more to families because child care is at least $150 a week, if not more. It would be nice to not have to work at nighttime all the time. It would be awesome to work in the daytime.

Such a setup, she says, would make working feel “worth the money,” since her earnings would not all be getting claimed by daycare costs.

Whitney, a Black mother in the Twin Cities, focuses on the need she sees for jobs adequate to supporting a family and for rental assistance:

To help them, the government would have jobs, because, like, we really don’t have any jobs available.... [H]ave one or two kids, give them a stable nice home. ... There’s lots of things the government should do. … Even for, like, single moms or moms with, you know, their kids...help them out more. Don’t, like, let them do it all themselves. Just to get [help] paying rent there. Help—they have to help more.

As mothers like Krista and Whitney thought about the needs of “families like theirs,” they articulated an array of resources that would help them make ends meet and care for their children.

Other mothers focused on different kinds of child-related needs, including more activities for children in their area and stronger schools. Rayna, a Black mother in New Orleans, compares what she sees around her Louisiana neighborhood to what she’s seen elsewhere; the activities and facilities she sees for children locally pale in comparison:

I think they could provide more activities for these kids to do. More safe activities and more better school systems. … Most neighborhoods have parks but like, it’s not always for the little kids. Like it should be for the little kids, too. ... And they do, but I’m saying more. And just more for them to do. Sometimes you go, like, out of town, you go out of Louisiana, and they have all kind of stuff for these kids to do and to play and to go to, you know, so I’m just saying we should have that here, too.

For Rayna, government responsiveness means providing children in her state with the same opportunities she sees other communities providing. Like Rayna, Kendra, a Black mother in New Orleans, also wants more attention to schools. “They should give the public schools more funding. They should give housing more funding.... Stop using the money for all the wrong reasons.” Kendra sees government taking action and allocating resources, but not as much to the types of supports she sees as necessary to meet families’ needs.

Finally, a few mothers focused less on the quantity of resources or how they were being allocated and more on the administrative hurdles required to access them. Houa, a Hmong mother in the Twin Cities explained:

And the government is already giving enough. … [T]he only thing that I don’t like is just paperwork, and paperwork, and paperwork, and just waiting on them, and waiting for the card, it’s my interview, and I have my interview, then they go over your case with you, you know? … Yes, like especially me with a busy life, I don’t have time to sit down and freaking do all the paperwork. It’s like, “Really?”

She says she jumps through these hoops because “I can’t just ignore it and not do it when it’s essential to the family,” but it feels like the government does not understand her needs as a parent when she is being asked to do so much while she is taking care of her children.

Houa is not alone in her experiences of administrative burdens. Over the course of our interviews with BEY: MV mothers, 94% shared at least one experience with administrative burdens (with many describing multiple instances), and their narratives spoke to the compliance, psychological, learning, and redemption costs they have faced. This high proportion of mothers reporting experiences of administrative burden is likely an undercount, as we did not systematically query all of mothers’ experiences over time and across programs. These experiences spanned income support programs, including but not limited to food assistance like SNAP and WIC (see, e.g., Barnes et al., 2023), voucher-based programs like Section 8 and child care assistance, and cash benefit programs like TANF and unemployment insurance. Mothers like Whitney recounted times when their benefits, like food assistance or cash welfare had abruptly stopped, often not knowing why, and the consequent struggle to get benefits reinstated as they sent in paperwork for verification or could not seem to get in touch with caseworkers. Some mothers described dedicating time and energy to long application and verification processes only to find out they had been deemed ineligible, though they were not sure why, or to have the disbursal of their benefits delayed. This was the case for Krista when she applied for food assistance; Whitney experienced this with child care assistance; and Kendra did when she filed for unemployment benefits.

Geographic variation in policy environments may influence how mothers think about and make claims on the government. Focusing on the claims that mothers more commonly discussed, we consider those child-oriented (e.g., schools and child activities) and financial resource-oriented (e.g., quantity of support, administrative burdens) in nature. Claims made by mothers from New Orleans were more frequently child-oriented than claims made by mothers in the Twin Cities (29% vs. 8%). Claims made by mothers in the Twin Cities were more frequently resource-oriented than claims made by mothers from New Orleans (45% vs. 27%). We are cautious in drawing any conclusions from this descriptive analysis due to our small sample size. However, it is possible that the Twin Cities offers more to mothers and their children than New Orleans (Kiernan, 2023), leading mothers in the Twin Cities to focus on financial resources in their claims on the government. Because our study occurs in the context of the cash transfer intervention, we also examine claims made by mothers receiving the large versus small BFY gifts. We do not find any differences.

Despite articulating an array of administrative burdens when they described their experiences with government assistance programs, the alleviation of these burdens was not the first place mothers’ thoughts went in discussing how the government could be responsive to families like theirs. Distinct from the focus of previous deductive research, mothers’ views of government did not solely reflect their experiences with burdensome targeted programs. For most mothers, burden reduction was not a salient domain for government responsiveness. Instead, mothers like Rayna, Kendra, Whitney, and Krista expressed the need for more generous resources to support parenting responsibilities and create opportunities for their children. They expressed a sense that these kinds of actions would indicate that the government cares for them and their families.

### Collective motherhood

Rather than referring to their identities as policy targets (e.g., WIC recipient) to express external political efficacy beliefs, respondents ground their views in a broader collective identity as mothers. Our question wording, which probed how the government could help “families like yours”—allowed mothers to speak more generally and not just about themselves. But as qualitative researchers know, if the framing we offer in our question wording does not align with participants’ views, they will often explicitly or implicitly reject that framing (for discussion of interview question development, see Castillo-Montoya, 2016). We see that the BFY: MV mothers did not typically do so, offering descriptions based in a sense of collective motherhood. We have 58 mothers describing 77 instances of desirable government responsiveness and, of these, 10 were centered on them as individuals and 59 expressed a collective sense of need.^4^

Our respondents regarded parenthood and the needs of children as deserving grounds for government response. We hear from Krista that “child care is expensive.” Note that she does not say it costs *her* too much—her statement is broader, as she argues that “they should offer more to families.” Her views reflect her belief in the necessity for government to respond to the needs of families with children broadly, rather than just to her family specifically. Similarly, Whitney discusses the needs of “single moms and moms...with their kids” as she articulates the need for better jobs and affordable housing. Rayna and Kendra also draw on this collective sense of needs, explaining that parks and schools need to be improved—their descriptions are not specific to the park or school in their particular neighborhoods. Rather, they present these as more general needs. This contrasts with Houa’s views, in which she describes the administrative burdens she faces in accessing government support as an individual experience, as opposed to a collectively shared one. The collective perspective expressed by mothers like Krista, Whitney, Rayna, and Kendra was most common across the BFY: MV mothers we interviewed. Because we hear a range of perspectives, we contend that the question wording may not have created the collectivism expressed in mothers’ responses.

It is not surprising that motherhood identity would be highly salient in respondents’ views of government, given the enormous role this identity played in moms’ lives. The mothers in BFY: MV described parenthood as a transformative experience, which aligns with findings from previous research (e.g., Edin & Kefalas, 2005). The transformative experience of motherhood was strongly and clearly articulated by many mothers, even though it was not something about which we explicitly asked. Deja, a Black mother in New Orleans, says, “[My baby], like, changed me tremendously in, like, so many ways. Like, I don’t even think I’m the same person because of my baby.” Patrice, a Black mother in New Orleans says:

I feel like my first time around, I mean it was unexpected when I had my first baby, but I feel like he changed me, like, as a woman, like you know. Like, I had somebody to really be responsible for 24/7. I had somebody who really loved me unconditionally for me....

Because motherhood is shaping so much of who our respondents are and how they see themselves, it serves as the identity basis for their external efficacy beliefs and the kinds of government responses they value. Our question about what the government could do for families like theirs evoked a deeply meaningful and critical source of identity for them. As political actors, therefore, motherhood—and collective motherhood for most—becomes a lens through which they interpret government responsiveness.

## CONCLUSION

Scholars have called for greater attention to the role of administrative burdens in creating policy feedback effects (Moynihan, 2022; Moynihan & Soss, 2014). At the same time, other researchers have encouraged the field to attend more to the perspectives of policy targets themselves (Barnes, 2020; Michener, 2018; Nuamah, 2022), calling for research that examines whether and how burdensome experiences shape the democratic lives of beneficiaries (Halling & Bækgaard, 2023). The present study addresses these questions using interviews with mothers with low incomes who have a good deal of experience interacting with multiple means-tested government programs, overcoming how a siloed approach studying experiences with discrete programs can miss the complex nature of the interactions policy targets have with various government programs simultaneously (Campbell, 2012).

We find that while experiences with administrative burdens are common and resented, these were not typically the most salient needs mothers referred to when describing needed areas of government responsiveness. Instead, drawing on a collective motherhood identity, the women pointed to needs such as family-friendly work, affordable child care, and local access to safe and engaging activities for children. Rather than being driven by an identity created by policy (in contrast with, e.g., Social Security’s creation of older adults as a politically relevant group [Campbell, 2003]), women were drawing on a culturally derived identity that then shaped their policy preferences. Were we to infer mothers’ external efficacy and needs for government responsiveness based on their descriptions of administrative burdens, we would fail to understand their actual political perspectives. While their experiences with administrative burden were widespread and frustrating, mothers did not usually present themselves as disempowered or lacking external efficacy. That they could make demands and saw their own demands as reasonable and appropriate seems to be based in the collective motherhood identity on which they draw in articulating their views of government responsiveness. While motherhood has been a politically sidelined identity in the study of administrative burden and policy feedback, our study suggests its salience among mothers with low incomes.

Our findings support previous research about the transformative experience of motherhood as shaping political perspectives (Greenlee, 2014). As Greenlee (2014) notes, “Being a mother often gives women a new vantage point on the political world...[and] provides them with new arguments, interests, and rationales for taking the political stances that they do” (p. 10). Rather than viewing themselves through the lens of a public assistance client, the women we interviewed expressed their expectations of the state through the lens of motherhood. Previous research catalogs the effective use of “motherhood” as a political strategy to attract women voters over time (Greenlee, 2014). Our findings suggest that motherhood appeals, especially when couched within expanded public provisions for children, may mobilize voters with low incomes.

Our study enriches the administrative burden literature by making a compelling case to explore gendered responses to administrative burdens. While studies suggest that parenthood is more influential in shaping the attitudes of women (Mercer, 2004), future research can examine how and when policy triggers a collective fatherhood identity through which men make sense of their experiences with the administrative state and politics. Studies can also examine whether experience with administrative burdens is a more salient “microcosm” of government for individuals without children than for those with them. In short, research can probe for whom administrative burdens and policy design matter most in shaping views of the state.

Future research can work to address some of the limitations of the present study. It is possible that mothers may have articulated their views differently had we phrased our question in a more individualistic way (e.g., “What more could the government do for you and your family?”). Also, we focus on the areas of government responsiveness that are most salient to mothers; this is not an exhaustive list of all areas in which mothers might desire government action. Like most qualitative work, the purpose of the present study is not to develop generalizable conclusions; rather, we focus on developing internally valid conclusions.

The present study highlights the merits of pursuing “bottom-up” research to understand more about administrative burdens and policy feedback effects (Michener et al., 2022) and looking across mothers’ multiple, simultaneous program experiences (Allard, 2009; Campbell, 2012). Despite common experiences with burdensome bureaucratic encounters, the most salient areas in which mothers articulated a need for government responsiveness tended to be greater resources to support the activities of parenting, like providing adequately for children’s material needs and offering access to stimulating and safe academic, care, and recreational environments. These desires are similar to those residents articulated in other communities with high levels of disadvantage (Barnes, 2020; Edin et al., 2023). Here, we see women who might be socially and politically marginalized by virtue of their gender, income, race, and reliance on means-tested government assistance largely displaying a sense of external political efficacy grounded in a collective motherhood identity.

These findings point to the need for inductive administrative burden research that considers political identities developed across a range of policies rather than based on a single public benefit. Doing so may reveal cumulative burdens or benefits that potentially shape views of government; this could motivate quantitative research to operationalize these realities in new ways. In addition, studies could consider benefit generosity as a form of administrative burden that shapes view of government. Meager benefits could constitute a salient “onerous experience” with policy, shaping program uptake overtime (Barnes et al., 2023) and how individuals assess government responsiveness (Campbell, 2003).

## Figures and Tables

**TABLE 1:** Characteristics of BFY: MV Mothers (*N* = 80).

	*N*	%	Median	Min	Max
At Wave 1					
Gift amount					
Large	40	50			
Small	40	50			
Site					
New Orleans	50	63			
Twin Cities	30	38			
Age					
Mother (in years)	80		27	19	42
Focal child (in months)	80		13	10	21
Race and ethnicity					
Asian	3	4			
Black	53	66			
Hispanic	7	9			
American Indian	1	1			
White	8	10			
Two or more races	7	9			
Other	1	1			
Motherhood experiences					
Mother’s number of children	80		2	1	6
Focal child is mother’s first child	23	29			
Romantic partner status					
Has romantic partner	45	56			
Co-resides with romantic partner	32	40			
Household size					
Full-time residents only	80		4	2	12
Lives with member of an older generation	25	31			
Across Waves					
Employment status					
Any formal employment	52	65			
Benefit receipt					
SNAP	≥70	≥88			
WIC	≥75	≥94			
Housing assistance	≥43	≥54			
Early care and education assistance	≥26	≥33			
TANF	≥26	≥33			
Program experiences					
Administrative Burden(s)	≥75	≥94			

*Note*: We provide Wave 1 demographic characteristic tabulations for context. We provide tabulations for formal employment across Waves 1 through 3, any previous and present benefit receipt across Waves 1 through 3, and experiences of administrative burden across all waves of interviews. Percentages are all calculated using the full sample size of 80 mothers as the denominator.

## Footnotes

1 Some mothers miss interview waves due to issues such as disconnected phones, incarceration, and other complex life events; the response rate for Wave 3 was 88% and 82% for Wave 4.

2 Estimates of participation are likely undercounts. We asked specifically about some income support programs (e.g., SNAP, WIC, TANF, housing assistance) across all waves; others were asked about at certain waves based upon the developmental stage of the study’s focal child (e.g., child care assistance) or came up in the context of overall income sources (e.g., SSI, UI). For mothers who have missed some interviews, we may not have captured their experiences as comprehensively.

3 Some mothers discussed more than one area in which they saw a need for government responsiveness. The areas that were only endorsed by one or two mothers included: the child support system, vaccines, programs for adolescents/emerging adults, US foreign policy, police, immigrant children without documentation, mental health, and racism.

4 The remainder did not describe areas of desired government responsiveness and so could not be classified.

## Abbreviations:

SNAP: Supplemental Nutrition Assistance Program
TANF: Temporary Assistance for Needy Families
WIC: Special Supplemental Nutrition Program for Women, Infants, and Children
